# Spherical hard carbon/graphite anode for high performance lithium ion batteries

**DOI:** 10.1371/journal.pone.0311943

**Published:** 2024-12-19

**Authors:** Xingqun Liao, Dalin Hu, Lijuan Yu, Bin Li, Feng Xiao, Shanxing Wang

**Affiliations:** 1 School of Chemistry and Materials Engineering, Huizhou University, Huizhou, China; 2 Huizhou Highpower Technology, Huizhou, China; Minia University, EGYPT

## Abstract

The issue of long charging time for electric vehicles has been a matter of serious concern, and the problem is mainly stemmed from the graphite anode. The slow kinetics of pure graphite can lead to the formation of the lithium metal during fast charging, which triggers cycle degradation and safety issues of electric vehicles. In order to ameliorate the fast charging issue, a spherical hard carbon/graphite porous electrode is devised. Based on this, the discharge capacity ratio at 3C shows an improvement of about 40% at 25°C and at 1C shows an improvement of about 18% at 0°C. Additionally, the 300-cycle capacity retentions exhibit increases of 12% and 14% at temperature of 25°C and 50°C, respectively. Generally, the analysis shows that the spherical hard carbon/graphite porous electrode has more uniform porous structure, shorter transport path, less nano-scale powder and a certain voltage buffer ability compared to the pure graphite powder system, which enhance the ion transport kinetics, and reduce the side reactions under the high temperature, so as to effectively improve the fast charging performance and cycle life of the LIBs. It is also proved that the kinetics improvement is not only attributed to the high kinetics inherited from the instinct of hard carbon, but also the porous electrode structures constructed by the two-size powder system of graphite and hard carbon.

## Introduction

The electric vehicle with the characteristics of high efficiency, clean, low carbon, is one of potential solutions to solve the energy issues, therefore large-scale promotion of electric vehicles will become an inevitable trend [1–7]. Lithium-ion batteries (LIBs) are widely used in electric vehicles because of their high energy density, long cycle life, no memory effect and other characteristics [8–11]. However, compared with traditional fuel vehicles, problems such as mileage anxiety and long charging time of electric vehicles seriously restrict their rapid development. Therefore, improving the fast charging of LIBs have become the goal of the electric automobile industry. Charging LIBs in a fast and safe way is mostly essential for the wide application of electric vehicles.

As the anode plays an essential role in fast charging, research on developing high-performance anode is being executed worldwide. Notably, three electrochemical storage mechanisms are involved in the anode materials: insertion/deinsertion, conversion, and alloying [1, 2]. Relying on the storage mechanism, several alkali metal ion anode materials, carbonaceous compounds, including soft/hard carbon and natural/synthetic graphite, are considered as the potential viable candidates. The carbon materials stand out as its low-cost, diverse structures, porosity, and global availability. Especially graphite has been the predominant commercial anode material for LIBs.

However, due to the slow kinetics of graphite anode, which is widely used, it is challenging to rapidly insert lithium ions [9–11]. At high rate charging, the graphite will generate serious polarization, low capacity and more side reactions, including lithium plating, thicker solid electrolyte interface (SEI) and a large amount of Joule heat [12–14]. During charging under low temperature, the maximum charging voltage and charging rate of the battery should be reduced to ensure the safety, otherwise lithium plating easily occurs. Many studies have shown [13–15] that the rapid charging of traditional LIBs is not affected by cathode deterioration or cathode surface film (CEI) growth, so lithium plating has gradually attracted the attention of researchers. In order to avoid these problems and achieve high-performance graphite for fast-charging LIBs, it is essential to accelerate lithium-ion kinetics and reduce its polarization.

In order to improve the kinetics of graphite, the industrial route is to make secondary particles through granulation, which can improve the isotropy of the particle system and effectively improve the charging rate of graphite [16]. However, pure secondary particles are easily to generate plastic deformation during calendaring process, leading to decreasing through holes and cross-linked holes. In this case, the conductive network tortuosity of the electrode is increased, which would cause severe local polarizations in the electrode and hamper cycle life [17–19]. Therefore, it is critical to develop a more efficient porous electrode structure [20–22].

In this paper, a structural design of the spherical hard carbon/graphite anode is studied. Except the high kinetics as previously reported [23], the spherical hard carbon with, weak plasticity, curved charging plateau as well as the low contact surface is utilized to synergistically improve the anode kinetics. The different proportions of spherical hard carbon/graphite anode were characterized by laser particle size analyzer (LPS), mercury injection apparatus (MIA), X-ray diffraction (XRD) and electron scanning microscope (SEM), to analyze the microstructure of porous electrodes. In addition, the electrochemical influence of mixture anode is also studied, illustrating the mechanism of improving the fast charge performance of the spherical hard carbon/graphite anode system.

## Materials and methods

### Preparation of slurry

Preparation of anode slurry: Spherical hard carbon (Japan ATEC) and graphite (Jiangxi Zichen Technology Co., LTD.) were mixed at 0:10, 1:9, 3:7, 4:6 and 10:0 to form active materials, and then mixed with CMC and SBR according to the weight ratio of active material: CMC: SBR = 98:1:1 in deionized water to make a homogeneous slurry. The resulting slurry was infiltrated by 200 mesh sieve for the following coating.

Preparation of cathode slurry: The cathode active materials were mixed according to the weight ratio of LCO: PVDF: Super P (SP) = 98:1:1 in NMP to make a homogeneous slurry. The resulting slurry was infiltrated with 200 mesh sieve for the following coating.

### Pouch cell fabrication

After cathode and anode slurries were prepared, extrusion coater was used to coat and produce the cathode and anode electrodes with the specified surface density, and the N/P ratio is 1.05. Then they were dried at 90°C for 15 minutes and then the electrode sheets were calendered to the desired thickness, cut to the desired sizes. The cathode, anode and separator of desired sizes were wound into jellyrolls and dried at 85°C in vacuum oven for 24 hours, and then sealed after liquid injection. Finally the cells were ready for electrochemical test after being aged at 45°C for 48 hours and then at 25°C for 24 hours.

### Electrochemical performance tests

The assembled soft pack batteries were tested on the battery detectors of model No LAND CT4008T of Xinwei Electronics Co., LTD. The voltage range was 3.0V-4.5V.

## Results and discussions

**Fig 1:**
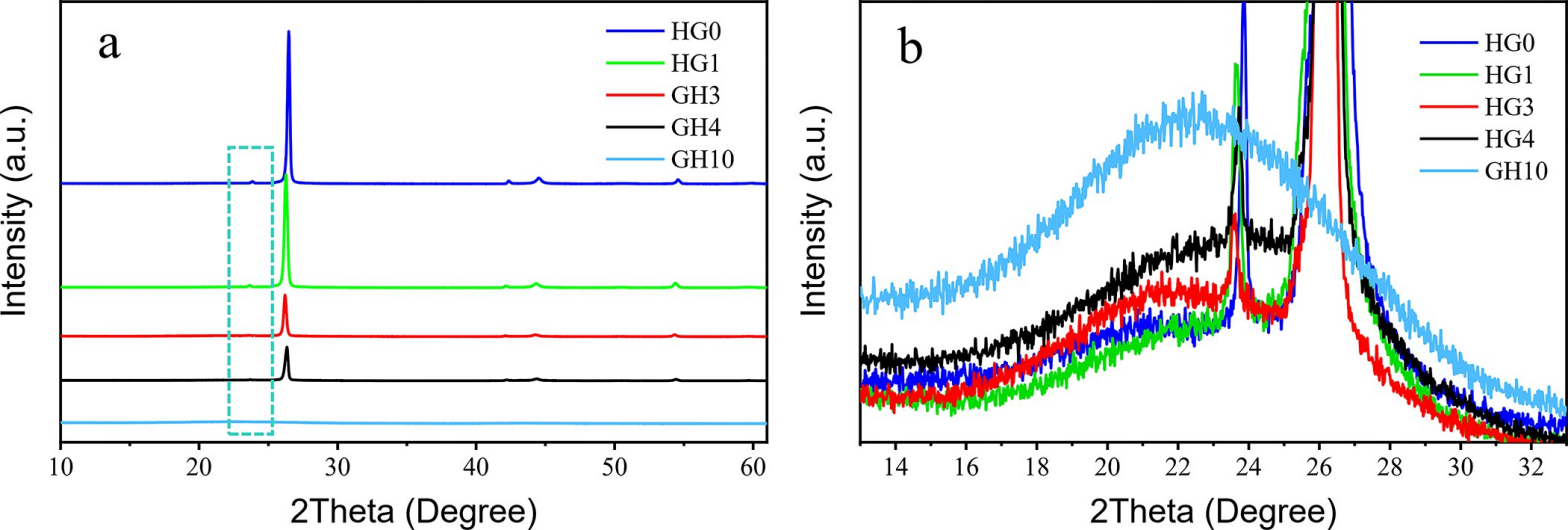
The full spectra of XRD (a) of HG0, HG1, HG3, HG4 and HG10; (b) the enlarged curves in the dashed box of Fig 1a.

After graphite and spherical hard carbon were mixed, XRD scanning tests were carried out on the five samples. HG0, HG1, HG3, HG4 and HG10 are denoted as varied mass ratios of spherical hard carbon and graphite powders (0:10, 1:9, 3:7, 4:6 and 10:0, respectively) in order to investigate the crystal difference of the hybrid powder system, as shown in Fig 1. The pure graphite material shows typical characteristic peaks at 26.24° and 44.26°, representing crystal facets of (002) and (004), respectively. When the hard carbon is added, defects such as microcrystalline units, carbon atom vacancies, layer spacing and carbon arrangement in hard carbon cause high-disorder structure, and thus the peak of (002) crystal facet presents an amorphous bulking peak, which is significantly different from the sharp peak of graphite (002) crystal facet, indicating the huge difference in microstructure of the two materials. In addition, the layer spacing of hard carbon is higher than that of graphite, thus the peak is shifted to the lower angle of 22.62° (Fig 1B). With the increase of the hard carbon ratio, the intensity of I(002) significantly decreases, and the value of I(004)/I(002) increases simultaneously, indicating that the graphitization degree of the hybrid powder system gradually decreases, stemming from the hard carbon increasing the disorder of the entire powder system, which is conducive to the improvement of the kinetics.

Owing to the different particle sizes of graphite and spherical hard carbon, the hybrid powder system is able to construct varied porous electrode structures. According to the ideal filling model, when the powder particles are spherical with a single size, the face-centered cubic packing and the hexagonal closest packing are the ones with the highest density, the largest coordination number and the smallest void. The hybrid system of graphite and spherical hard carbon is similar to the single particle powder filling model but with the small-size particles filling into the voids around big practices. According to the Hudson filling model of the two-size powder filling system, when the size ratio of two particles is 0.1716, the small size particles is able to fill the triangular pores constructed by the big practices, resulting into the most compact particle system. By LPS test, the particle size distributions of graphite and spherical hard carbon are shown in Table 1. Although the actual particle size, distribution, shape, surface roughness and inter-particle force of the powder are different, and the actual filling situation will become more complicated [24–26], here we can roughly calculate the packing effect of the powder through the Dv50 of the powder. It can be seen from Table 1 that the Dv50 of graphite and spherical hard carbon are 13.07 and 1.80 μm respectively. It can be calculated that Dv50_spherical hard carbon_/Dv50_graphite_ = 0.1377, which is lower than the minimum particle size ratio of Hudson’s the most compact packing model, indicating that the spherical hard carbon particles can be fully filled into the triangular holes stacked by three graphite particles, forming a compact particle packing system without changing the compact framework of big particles. Therefore, this ratio of graphite and hard carbon particle size can form a good void filling, and can play an important role in supporting and regulating the void of the hybrid powder system.

**Table 1:** The particle size distributions of graphite and spherical hard carbon.

Name/parameter (μm)	Dv100	Dv99	Dv90	Dv50	Dv10	Dv0
graphite	31.00	27.08	20.74	13.07	8.09	4.74
spherical hard carbon	38.60	18.65	4.20	1.80	0.88	0.49

**Fig 2:**
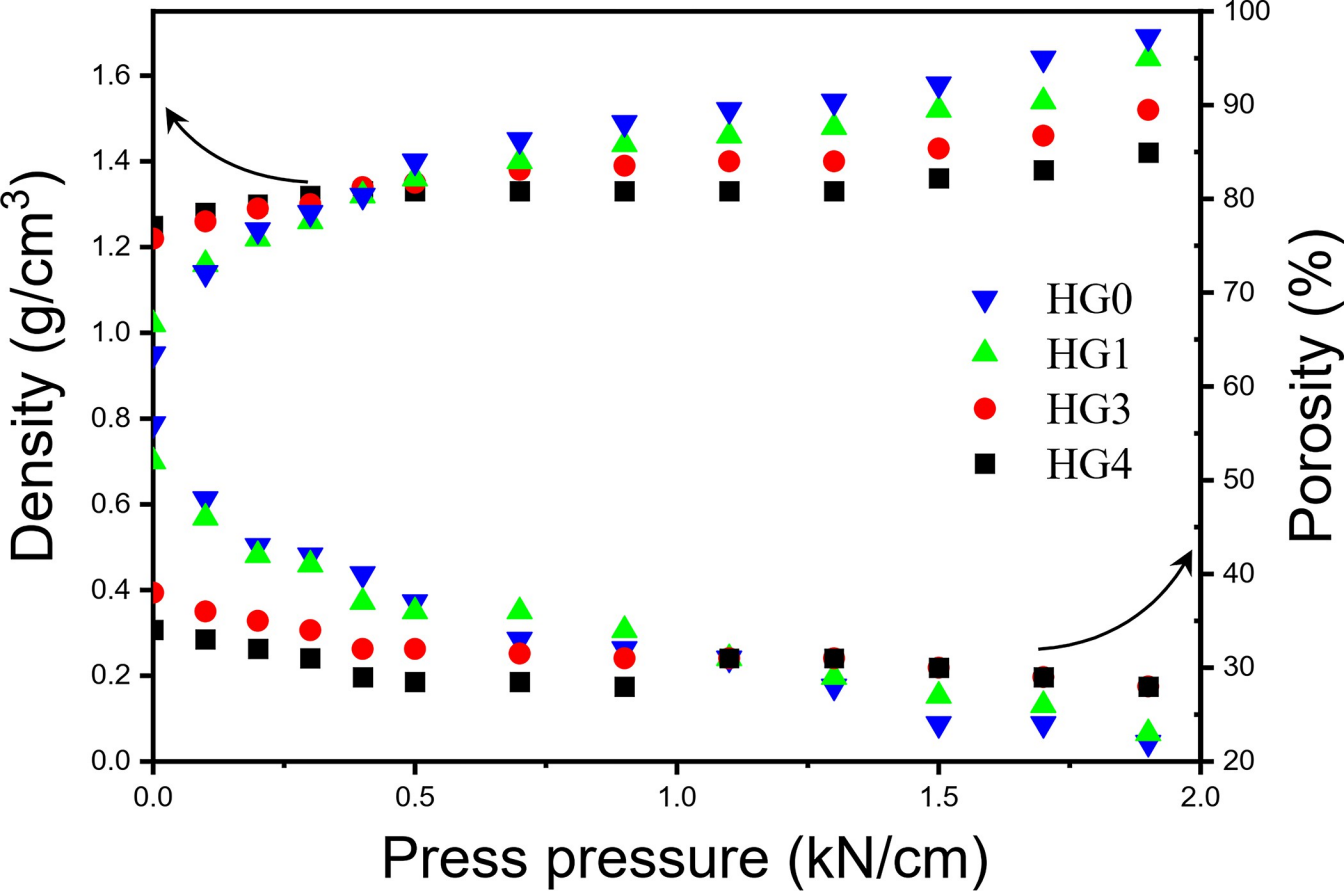
The porosity and density variations of HG0, HG1, HG3 and HG4 under different pressures.

From the above theoretical calculation and speculation, the addition of hard carbon to graphite will effectively change the powder and void distributions. In order to explore the void regulation rules of actual powder, the density and porosity tests were conducted on powders of different proportions. According to the data shown in the Fig 2, at a lower pressure (< 0.4 kN/cm), the compaction of the blended powder system increases with the addition of hard carbon, and the void at this stage also decreases until the pressure reaches 1.1kN/cm, which is consistent with our theoretical speculation. It shows that the addition of small particles is conducive to the void filling of the whole powder system and supporting the whole powder system. When the pressure applied is higher than 1.1 kN/cm, with the increasing amount of hard carbon, the variation of compaction density and porosity have the opposite rule. The increments of compaction density gradually decrease and the decrements of porosity increase, compared to the pure graphite powder system. It’s mainly because the plasticity of hard carbon particles is lower than that of graphite secondary particles. Graphite secondary particles tend to generate more deformation under the increasing pressure and less rebound after releasing, however, the spherical hard carbon is more resilient, which enables the powder system generate more voids, forming evenly dispersed void in electrodes.

**Fig 3:**
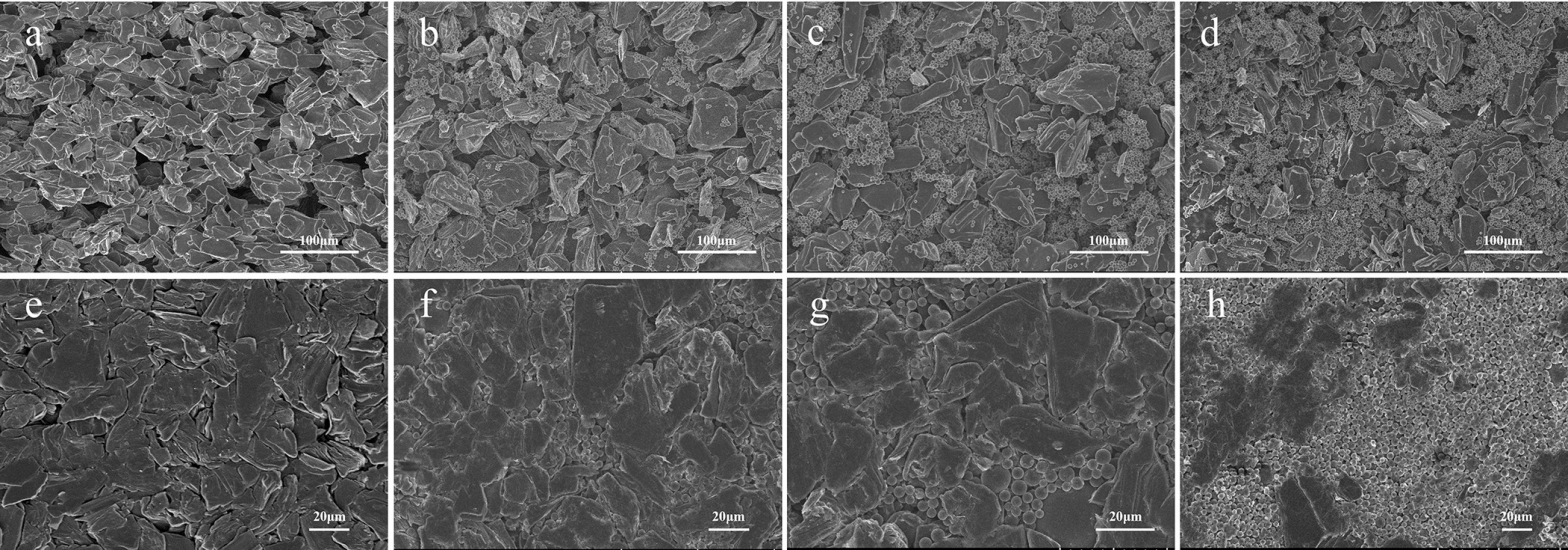
The SEM images of coated (a-d) and calendered electrodes (e-f); (a, e) HG0, (b, f) HG1, (c, g) HG3, (d, h) HG4.

In order to visually display the powder system distribution of spherical hard carbon/graphite, the coated and calendered electrodes with different proportions of spherical hard carbon and graphite are compared by SEM in Fig 3. The morphology of coated pure graphite electrode shows different sizes of voids (Fig 3A) in the powder system. In the hybrid powder system, the hard carbon particles are randomly filled into the voids between the graphite. As the proportion of hard carbon particles increases, void filling becomes more dense, and the size and distribution of voids throughout the system become more uniform (Fig 3B and 3C). However, when the spherical hard carbon proportion reaches 40% (HG4), the hard carbon particles are surplus, and some of the particles overflow the void to cover the surrounding graphite (Fig 3D). This filling ratio rule is consistent with the results of Tanaka et al.’s study on the actual two-size powder filling. The study indicates that as the volume fraction of small particles increases, the filling rate rises linearly and it reaches the maximum when the volume fraction of small particles is 29%. If small particle quantity continues to increase, the filling rate decreases linearly, indicating that the voids between graphite cannot accommodate more small particles [27, 28].

The void-distribution rule of the powder system becomes more obvious after calendering (Fig 3E–3H). For the calendered pure graphite electrode, the graphite powder spacing is compressed, and the voids become smaller or even disappear. Specifically, the large voids become smaller and the small voids disappear, resulting in a sharp decrease in through holes and cross-linked holes. This is mainly because the main body of commercial graphite is secondary particles, plasticity of which is high, so the deformation is hard to recover after rolling, leading to the seamless contact between graphite particles. This seamless contact will cause the electrolyte impossible infiltration, increasing the tortuosity of ion transport path, and thus deteriorating the kinetics (Fig 3E). In the hybrid powder system, although the space between graphite and spherical hard carbon is compressed, majority of through holes and crosslinking holes still remain. Specifically, the 1/9 spherical hard carbon/graphite blend ratio shows that, in the areas without spherical hard carbon, the graphite is compressed, forming the seamless contacts as same as the situation observed in the pure graphite electrode. On the contrary, in the areas with the spherical hard carbon, the spherical hard carbon fills the voids of the graphite, playing supporting roles to generate an even void distribution. Moreover, the strong resilience of spherical hard carbon exhibits a strong recovery ability, so the even void distribution in hybrid powders is possibly retained after pressing. Additionally, it is due to the spherical morphology of hard carbon, the contact between the particles is point contact, which is less possible to stick together and form seamless interfaces. For the HG3, it can be seen that the voids between graphite are reasonably filled, and form a porous electrode with uniform-distribution void structures, which enables to promote the penetration of the electrolyte, resulting to shortened the tortuosity of ion transport path, and improved the kinetics of the electrodes. However, for HG4, the excessive spherical hard carbons slip out the voids and cover the graphite surfaces after calendering. In this case, the powder system will change from the hard carbon/graphite hybrid system to two independent powder systems. Although the void ratio is still high, the uniform void structure of the porous electrode disappears, resulting in losing the high kinetics constructed by the hybrid powder system.

**Fig 4:**
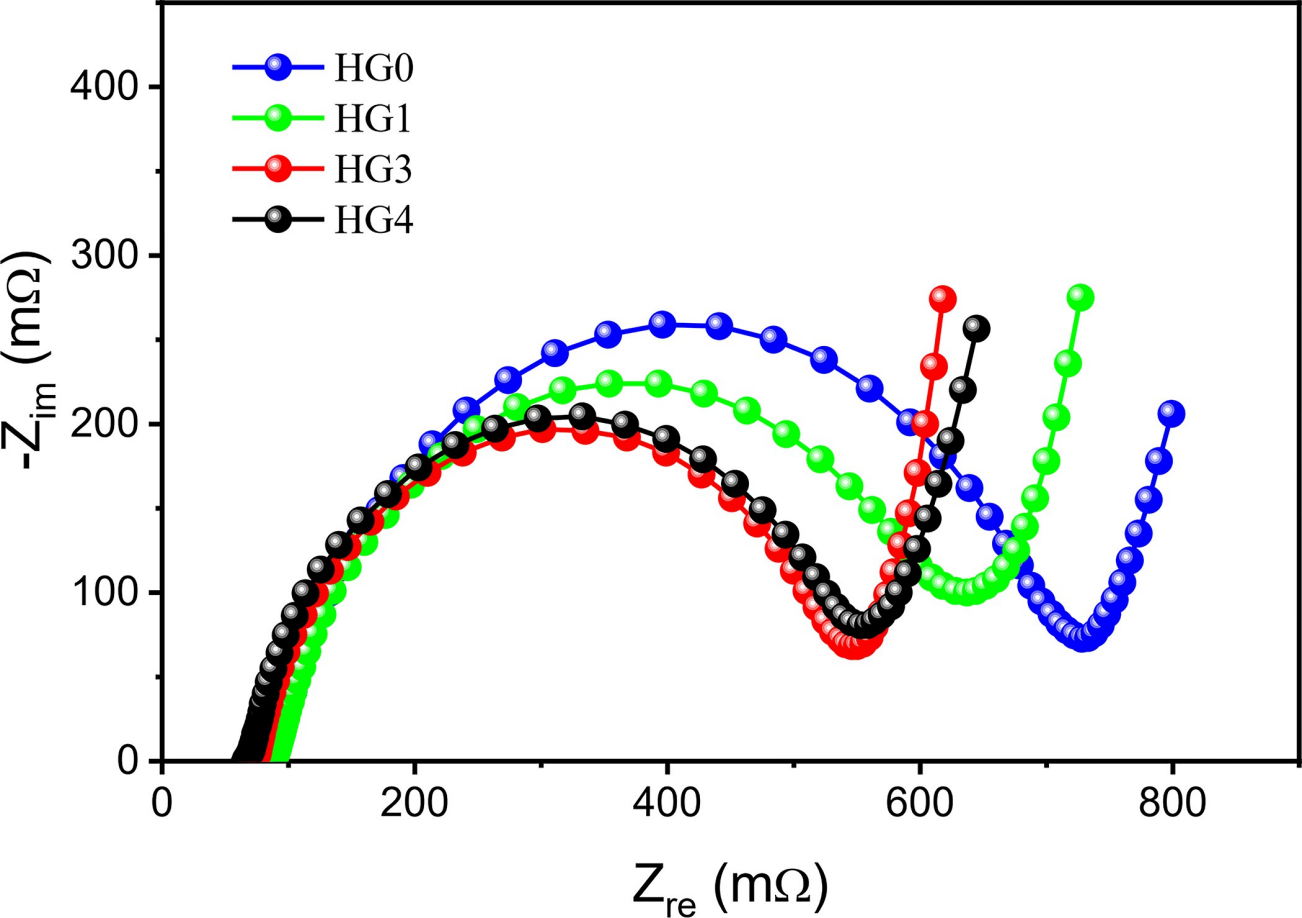
The EIS curves of HG0, HG1, HG3 and HG4.

In order to compare the kinetics of pure graphite system and hybrid powder system, pouch cells were assembled. To fit the actual situation in factories, the compaction density of the cell was designed to 1.6g cm^-3^, which was close to the ultimate compaction density of the HG4. The EIS test results are shown in Fig 4. It can be seen that with the increase of the proportion of hard carbon, the charge transfer impedances of the batteries gradually decrease, which on the one hand is due to the high kinetics brought by the intrinsic isotropy and the large layer spacing of hard carbon; on the other hand, the charge exchange is accelerated by the larger contact area between the active materials and the electrolyte under the proper compaction density [29, 30]. For HG4, its impedance shows inferior to that of the HG3, which indicates that the ion transporting paths are declined, and the advantages of the mix powder system disappear in HG4, well consistent with our previous results.

**Fig 5:**
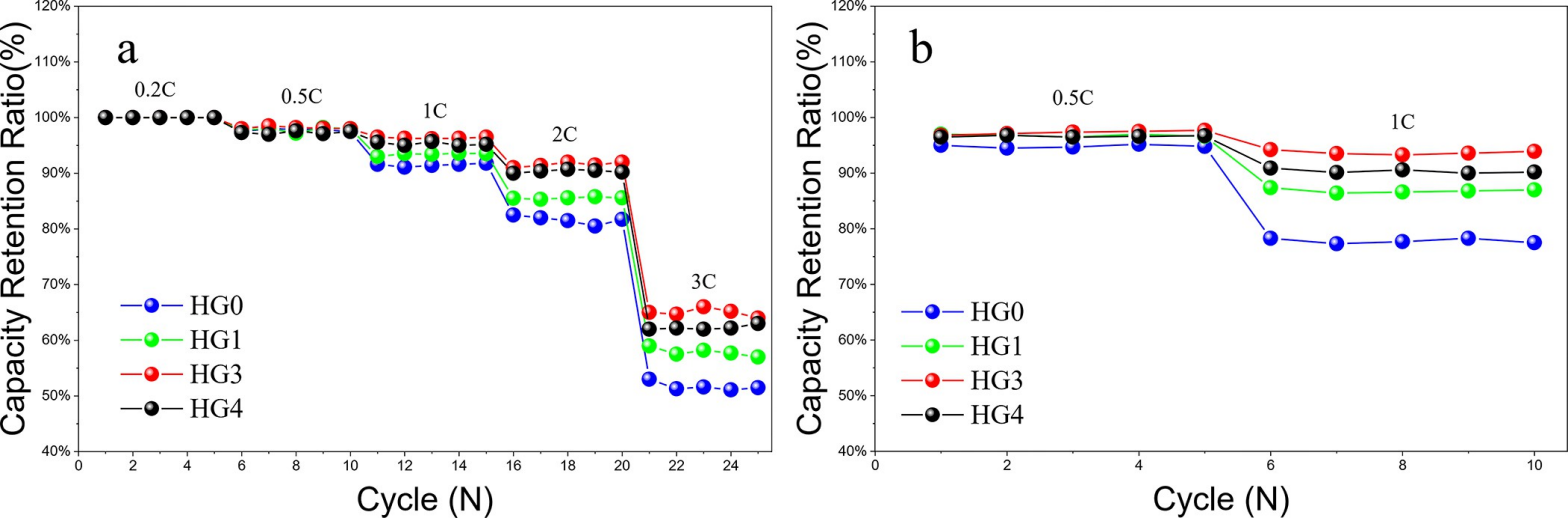
The rate performances of HG0, HG1, HG3 and HG4 running with 0.5C, 1C and 2C at room temperature and with 0.5C and 1C at 0°C.

To investigate the influence of the spherical hard carbon/graphite powder system on the rate performance, the LIBs were charged and discharged under different current densities, as shown in Fig 5. It can be seen from the rate curves that the addition of spherical hard carbon benefits to the rate performance, which is consistent with the EIS results. At low charging rate, the performance differences between samples are not obvious, but with the increasing current densities, the mix powder specimens exhibit better performances. At 3C, the capacity retention of HG3 is about 10% higher than that of HG0. The reason is that, on one hand, the high-rate instinct of hard carbon promote the kinetics of the entire powder system and on the other hand, the small particles of hard carbons fill into the graphite voids to support the entire powder system, forming a uniform porous electrode structure, reducing the tortuosity of the conductive network. As to the HG4, the rate performance decreases, which is consistent with our previous conclusions. It can also be seen that the improvement of rate performance is not only related to the high kinetics of hard carbon, but also the effective filling of different-size particles.

**Fig 6:**
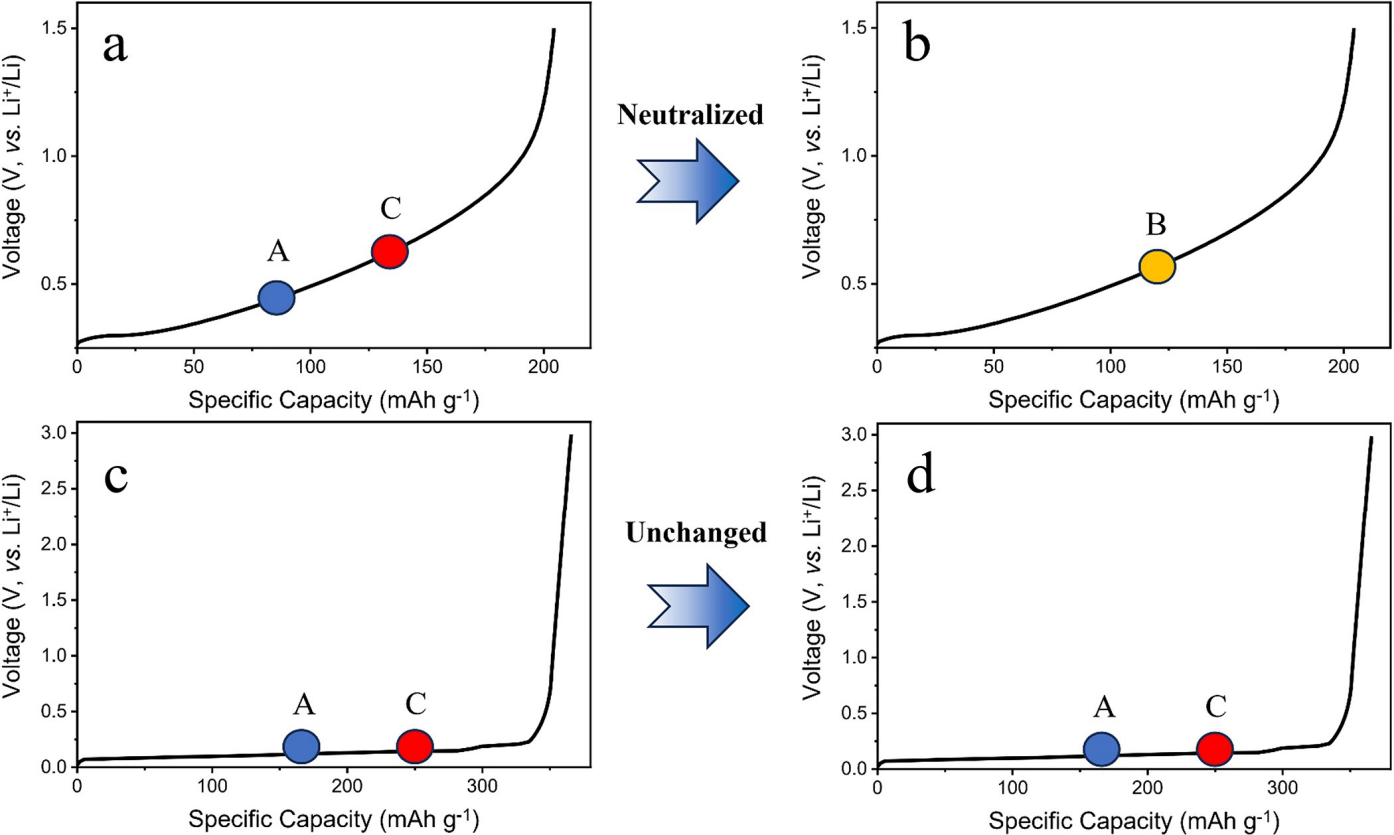
The illustration of charging-platform differences between hard carbon (a, b) and graphite (c, d).

For a further study about the rate enhancement mechanism of the mix powder system, the rate tests of HG0, HG1, HG3 and HG4 at low temperature were carried out. Short cycle tests of 0.5C and 1C were implemented at 0°C. The capacity retention equals to the tenth-cycle capacity divided by the second-cycle capacity, as shown in Fig 5B. At 0.5C, there are small gaps between each specimen, within 2%. At 1C, big gaps occur. The HG0 exhibits the lowest capacity retention of about 78%, whereas the performance of the HG1 is significantly improved, reaching about 86%. For the HG3, the capacity retention is further increased to about 94%. Interestingly, comparing the results of 1C at 0°C and 25°C, it implies that the capacity retention of HG0 at 0°C is 14% lower than that at 25°C, whereas the capacity retentions of HG1, HG3 and HG4 at 0°C are merely 6%, 4% and 5% lower than those at 25°C. The capacity improvement at 0°C of hybrid power system is mainly contributed to the charging plateau difference between the hard carbon and graphite. To be specific, the hard carbon charging plateau is a sloping type, which leads to the local potentials being neutralized to a balanced state. As illustrated in Fig 6A and 6B, the potentials of A and C tend to neutralize to the potential of B, which is conducive to ameliorating lithium plating, thus improving the low-temperature performance. However, the graphite plateau is flat, impossible to neutralize local potentials.

**Fig 7:**
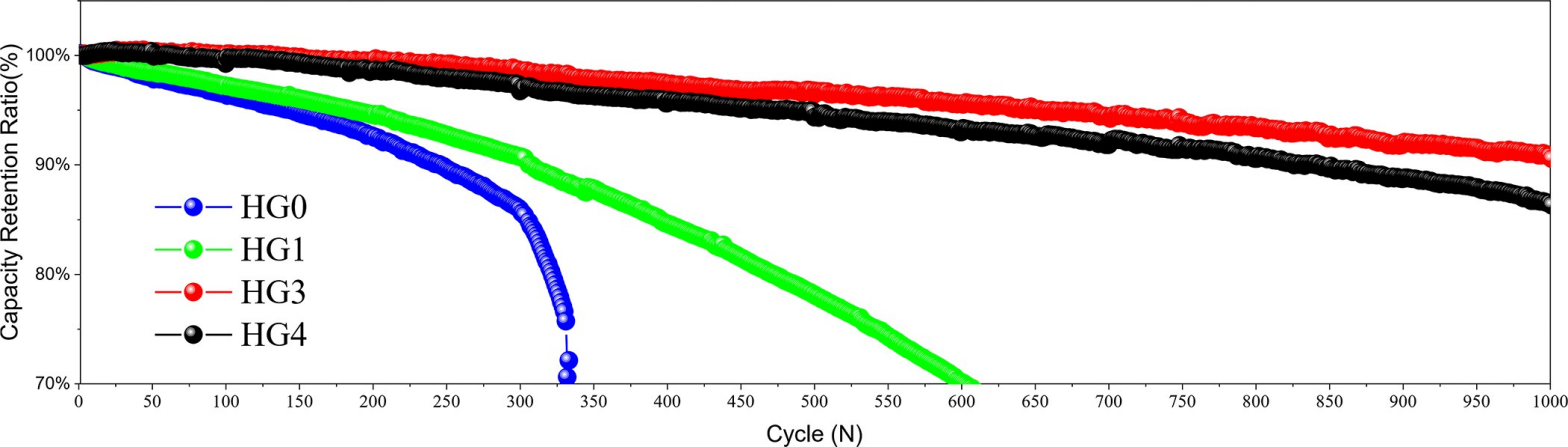
The cycle performances of HG0, HG1, HG3 and HG4 at room temperature.

In order to study the influence of the hard carbon/graphite system on the cycle ability, the LIBs were carried out the cycle tests at 1C under 25°C, the voltage window was 3–4.5V, and cut off at 0.05C. The HG0, HG1, HG3 and HG4 display initial coulombic efficiencies (ICEs) of 90.5%, 89.3%, 87.1% and 86% respectively, which follow a linear relationship as a function of the hard carbon content (S1 Fig). According to the data in S2 Fig, the coulombic efficiencies during cycling at 25°C remain almost 100% except the first few cycles. The cycling results in the Fig 7 are as expected. For the pure graphite sample of HG0, the capacity retention shows the lowest of 86%. However, for the rest of samples with hard carbon, the cyclic capacity retentions are obviously improved. For the HG3, the cycle capacity retention reaches the highest one of 98.5%. But for the HG4, the capacity retention slightly deteriorates to 97.5%. The main reason is that as the compaction density is close to its limit, the hard carbon filling effect is weakened, gradually deteriorating the electrolyte penetration and ion conductivity, although the hard carbon is of intrinsically high kinetics. Besides, according to the data in S3 Fig, adding the hard carbon is also beneficial to the cycling swelling control at the temperature of 25°C due to the improved kinetics and the declined risk of lithium plating.

**Fig 8:**
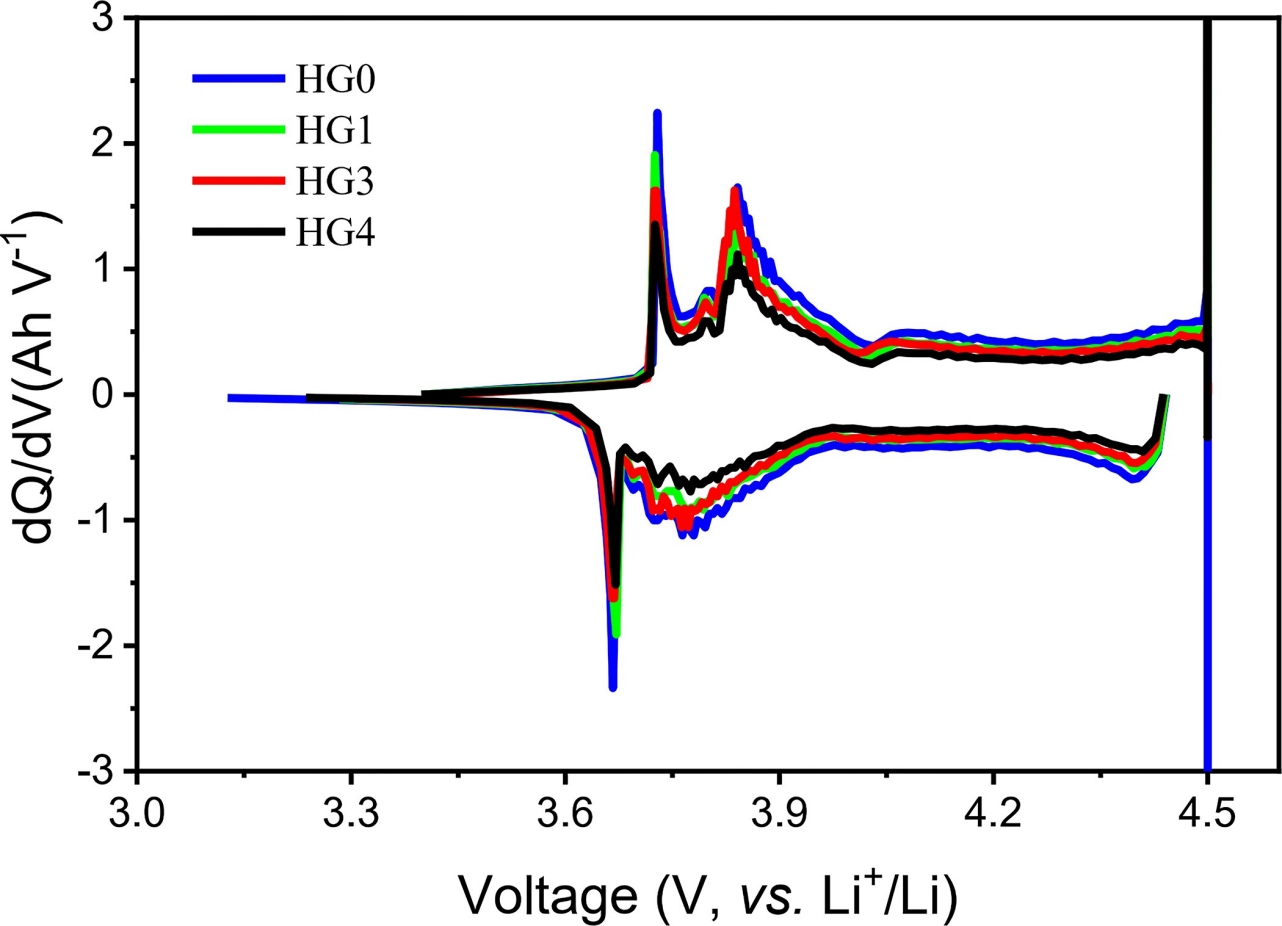
The dQ/dV curves of HG0, HG1, HG3 and HG4.

In order to further study the improvement mechanism of spherical hard carbon/graphite on the cycle, the batteries were charged and discharged at constant current and constant voltage at 0.1C to 4.5V until the current was 0.02C, and then discharged at 0.1C to 3V for 3 cycles. The dQ/dV analysis was performed by using the second cycle data, as shown in Fig 8. It can be seen that the dQ/dV curves of all samples consist of two oxidation peaks (3.73V and 3.83V) and two corresponding reduction peaks (3.67V and 3.77V). With the increasing amount of hard carbon, the peak intensities of oxidation and reduction of dQ/dV curves decrease gradually. This decreased kinetics of lithiation and delithiation reactions is able to effectively weaken the concentration polarization of anode. Meanwhile, based on the previous results, the hard carbon/graphite hybrid system enable to promote the mass-transfer kinetics of the porous electrode. Therefore, these two processes are slowed down and improved respectively, which can smooth out the differences of each process and promote the reactions to be homogenized, weakening the local polarization, and thus improving the room temperature cycle performance.

**Fig 9:**
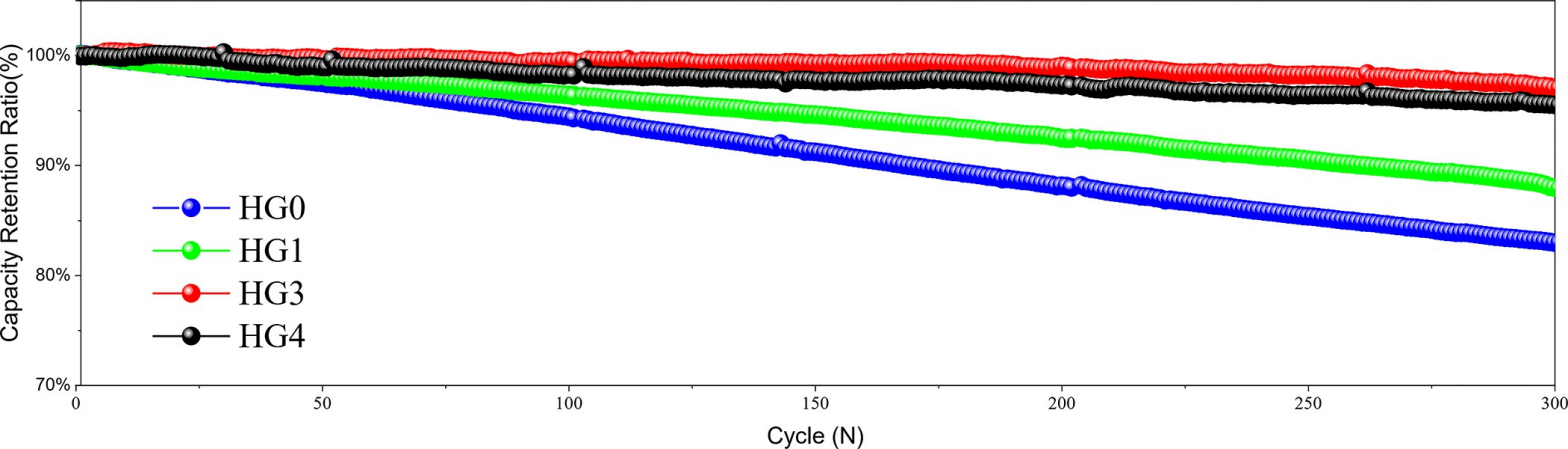
The cycle curves of HG0, HG1, HG3 and HG4 at 50°C.

3C products are usually affected by temperature fluctuations in the daily life, especially high temperature cycling, thus the cycling test at 50°C was implemented. Test conditions are as follow: 1C constant current then constant voltage charge to 4.5V, cutoff at 0.05C, and then discharge to 3V with 1C. The ICEs at the temperature of 45°C exhibit about 1% lower than those at the temperature of 25°C. It’s due to the more reactions to generate thicker SEI at the high temperature. According to the data in S4 Fig, the coulombic efficiencies during cycling at 45°C remain almost 100% except the first few cycles as same as cycling at 25°C. As shown in Fig 9, the HG0 exhibits the worst performance, with a 300-cycle capacity retention of about 83%. In the hybrid powder samples, the high temperature cycle performances are obviously improved. HG3 exhibits the best performance and the capacity retention reaches 98%. As for HG4, the high-temperature cycle advantage of the hybrid powder system no longer exists. The improvement of high temperature performance is mainly due to the different preparation process of hard carbon and graphite. The hard carbon preparation does not have a crushing process like graphite, so that no nano-scale powders are introduced. Nano-scale powders have large specific surface areas and are highly active, easily reacting with electrolytes to produce gases at high temperature, resulting in poor contact between active materials and the copper foil, which seriously deteriorates the cycle performances. It is also inferred that although the average particle size of hard carbon is around 2μm, the side reaction effect at high temperature is negligible, far less than the side reactions caused by graphite nano-scale powders. In addition, there is no obvious impact on swelling by adding hard carbon for cycling at the temperature of 45°C (S5 Fig).

In order to check the structural stability, the 1000-cycle at room temperature and 300-cycle at 45°C HG3 were disassembled and the cycled electrodes were scanned by XRD. According to the spectra in S6 Fig, there is no structure changes since all peaks of the cycled batteries are still well matched with that of fresh HG3. The decreased intensities of peaks are contributed to the SEI covered on the batteries surfaces. The minor peaks appeared in the high temperature cycling data are originated from the lithium hydroxide. This may be caused by the lithium plating or not being discharged completely.

## Conclusions

In this paper, we have demonstrated spherical hard carbon/graphite hybrid system to optimize the structure of porous electrode and the kinetics of battery system. Our analysis indicates that the HG3 provides the maximum performance with the set of ratios tested. For HG3, we have demonstrated the capacity retentions are 40% larger at 3C under room temperature and 18% larger at 1C under 0°C than those of batteries using graphite anodes under the same conditions. Additionally, HG3 shows the remaining capacities at 1C after 300 cycles are 12% larger at room temperature and 14% larger at 50°C. Therefore, the hybrid anode design significantly improves fast charging performances.

The physical property analysis shows that with a small proportion of hard carbon, the compaction density and porosity of the hard carbon/graphite system increases and decreases respectively. The EIS result exhibits that the charge transfer speed of the hybrid powder is improved, which is proofed by SEM characterization. It indicates that the added hard carbon particles fill the voids between graphite particles. As to the hard carbon, on the one hand, it plays a supporting role, reducing the adhesion of graphite interfaces, and forming a porous electrode structure with a uniform-void distribution. On the other hand, it acts as a conductive media to conduct electrons, reducing the tortuosity of the system, and improving the charge conductivity of the system. By comparing the fast-charge capacity retentions under 25°C and 0°C, it implies that owing to the curved plateau of hard carbon, the different voltages in the electrodes could reach to a neutralized voltage, which is conducive to ameliorating the lithium plating. The further study implies that the introduction of hard carbon enables to weaken the dQ\dV redox peaks, indicative of slowing down the lithiation and de-lithiation reactions to weaken the concentration polarization, leading to the reactions of the whole system more homogenous, thus improving the cycling performances. The high temperature cycling results show that the preparation of hard spherical carbon does not have the crushing process as required for graphite, so it does not contain nano-scale powder, resulting in less side reactions under high temperature and thus obtaining the better cycle performances under high temperature than the LIBs of pure graphite.

In general, the spherical hard carbon/graphite mixture technology can effectively improve the fast charging and cycling performances, and has a significant guiding for the structure design of porous electrodes.

## Supporting information

S1 Fig The initial coulombic efficiencies of batteries cycling at 25°C and 45°C. (DOCX)

S2 Fig The coulombic efficiencies of HG0, HG1, HG3 and HG4 cycling at room temperature. (DOCX)

S3 Fig The thickness swellings of HG0, HG1, HG3 and HG4 cycling at room temperature. (DOCX)

S4 Fig The coulombic efficiencies of HG0, HG1, HG3 and HG4 cycling at 45°C. (DOCX)

S5 Fig The thickness swellings of HG0, HG1, HG3 and HG4 cycling at 45°C. (DOCX)

S6 Fig The XRD spectra of the fresh, 1000-cycle at 25°C and 300-cycle at 45°C HG3. (DOCX)
