# Supplementary material for: Spherical hard carbon/graphite anode for high performance lithium ion batteries
Source: PLoS One. 2024 Dec 19;19(12):e0311943. doi: 10.1371/journal.pone.0311943 (PMC11658506; doi:10.1371/journal.pone.0311943)
Supplement: S3 Fig — (DOCX) [file pone.0311943.s003.docx]

***Spherical hard carbon/graphite anode for high performance lithium ion batteries***

**Xingqun Liao^1, 2^, Dalin Hu^2^, Lijuan Yu^2^, Bin Li^1^, Feng Xiao^1*^, Shanxing Wang^1*^**

1. School of Chemistry and Materials Engineering, Huizhou University, Huizhou, China

2. Huizhou Highpower Technology, Huizhou, China

* [csuxiafeng@163.com](mailto:csuxiafeng@163.com); wsx030700@163.com


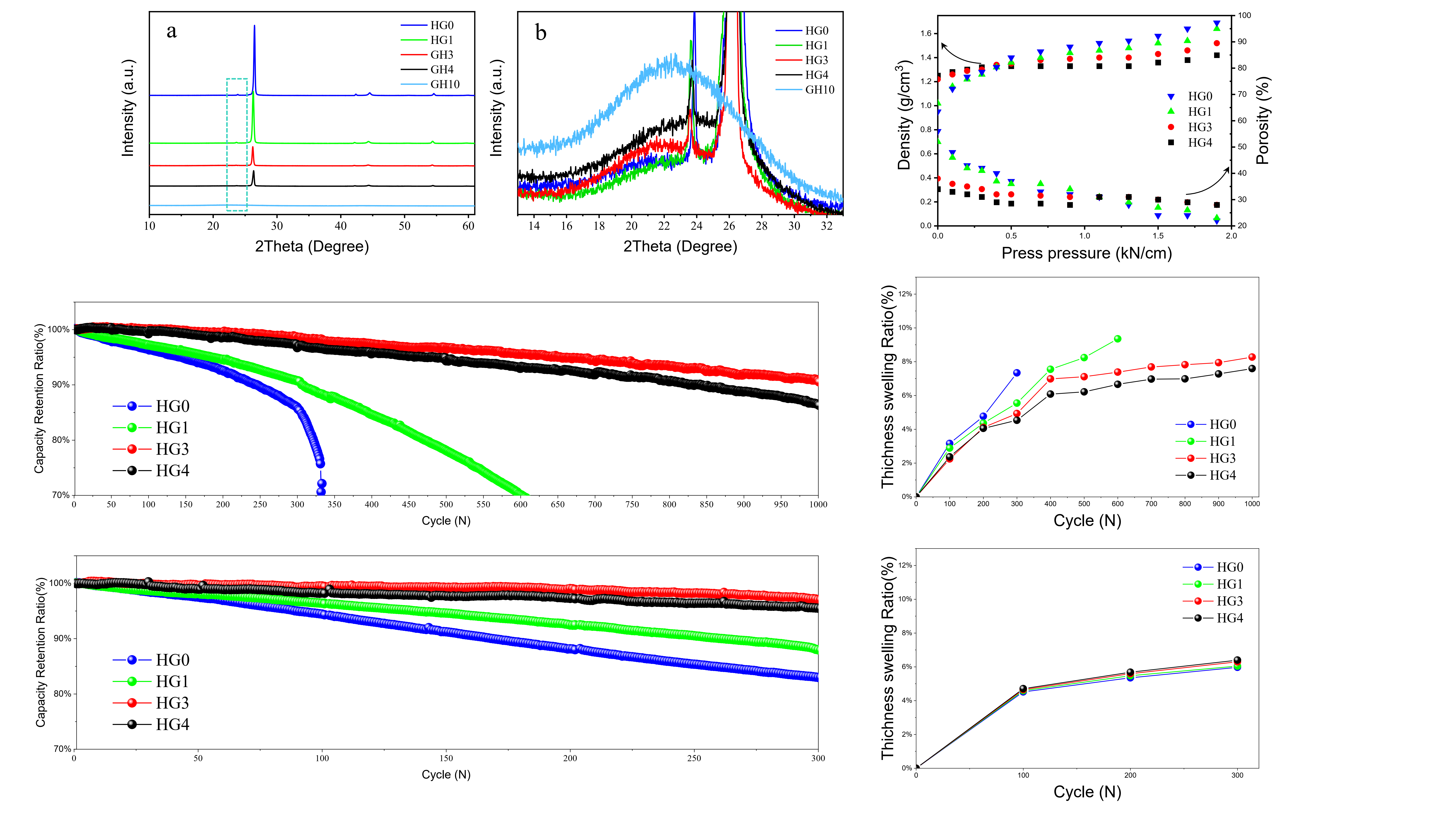


S3 Fig. The thickness swellings of HG0, HG1, HG3 and HG4 cycling at room temperature.
